# Determining Breast Implant Prevalence: A Population Study of Italian Chest Radiographs

**DOI:** 10.1007/s00266-023-03290-6

**Published:** 2023-02-24

**Authors:** Fabio Santanelli di Pompeo, Guido Firmani, Guido Paolini, Mark Warren Clemens, Giuseppe Argento, Giulia Marta Barelli, Elisa Rosati, Claudia Zanovello, Gennaro D’Orsi, Michail Sorotos

**Affiliations:** 1 Department NESMOS-Sant’Andrea Hospital, Faculty of Medicine and Psychology, Sapienza University of Rome, Via di Grottarossa 1035-1039, 00189 Rome, Italy; 2 Department of Plastic Surgery, M.D. Anderson Cancer Center, 1400 Pressler St., Unit 1488 Houston, Texas, 77030 USA; 3 Radiology Unit, Department of Medical-Surgical and Translational medicine, Sapienza University of Rome-Sant’Andrea Hospital, Via di Grottarossa 1035-1039, 00189 Rome, Italy

**Keywords:** Breast implants, Implant prevalence, Chest radiographs, Implant epidemiology

## Abstract

**Background:**

Current breast implant prevalence within the general population remains elusive. An accurate prevalence is critical to serve as the denominator for any assessment of breast implant-related complication. The purpose of this manuscript is to assess this prevalence in women aged 20–70 years in Italy.

**Materials and Methods:**

Eight reviewers, demonstrating a mean sensitivity of 87.0% and specificity of 97.0%, were recruited for retrospective identification of implants on chest radiographs from a tertiary academic hospital in a major urban setting. Three final reviewers were selected, and they assessed all eligible chest radiographs collected between January and December 2019. The hospital-based population was compared to epidemiological data at a local, regional and national level to demonstrate homogeneity of age structures using the phi correlation coefficient.

**Results:**

We identified 3,448 chest X-rays which yielded 140 implants, with an overall prevalence of 4.1% for women aged 20–70. Implants were bilateral in 76% of cases and unilateral in 24%. They were placed cosmetically in 47.1% cases and used for reconstruction in 52.9% cases. Phi correlation coefficient found no differences across hospital-based, local, regional and national populations.

**Conclusion:**

A validated method was performed to estimate implant prevalence from an academic hospital in a major urban setting at 4.1% and was used to estimate national prevalence in Italy. The implications of this epidemiologic study may reach across national borders for improved understanding of breast implant epidemiology and in predicting the total number of patients within a given population that may be affected by device complications.

**Level of Evidence IV:**

This journal requires that authors assign a level of evidence to each article. For a full description of these Evidence-Based Medicine ratings, please refer to the Table of Contents or the online Instructions to Authors www.springer.com/00266.

## Introduction

The US Food and Drug Administration recently issued several orders to strengthen breast implant (BI) risk communication including restricting implant sales, boxed warnings, mandatory informed consent checklists, and updated surveillance recommendations [1, 2]. In 2021, the scientific committee on health, emerging and environmental risks (SCHEER) of the European Commission released a detailed review of breast implant safety and updated guidance on breast implant associated-anaplastic large cell lymphoma (BIA-ALCL) [3]. An identified gap within the scientific literature was an accurate assessment of national and global breast implant prevalence critical for the determination of complication rates and the extrapolation of clinical outcomes from post-market approval trials. As a result of mass media coverage and public scrutiny, considerable research efforts have been directed toward understanding diseases related to BI, including BIA-ALCL [4, 5]. Despite the numerous efforts, several points remain unanswered, including the BI prevalence in the general population. This represents the denominator necessary to calculate accurate rates of any BI-related sequelae, important to physicians, manufacturers, government authorities, and patients assessing the risks and benefits of these devices.

As a consequence of lack of mandatory National Breast Implant Registries (NBIR) and only few being opt-out, epidemiologic studies on BI prevalence in the scientific literature are few and sparse [6]. Traditional estimation methods include sales reports from implant manufacturers, surgery reports from registered surgeons affiliated with plastic surgery societies, mail-based surveys, and subjective expert opinion [7–10]. Though straightforward, these methods are flawed, use incomplete datasets and provide inaccurate estimates [11]. Once established, NBIRs will take long before collecting useful data, allowing for unconventional alternatives to emerge. De Boer et al. [12] conducted a study design identifying radiopaque BIs on chest radiographs (Chest X-ray or CXR) to calculate BI prevalence within the Dutch female population. We intended to validate their results by using the same methodology in Italy, a country with a larger female population than The Netherlands (30.8 vs. 8.75 million), and 5th worldwide for cosmetic breast augmentation surgeries [13]. BI prevalence was determined as primary endpoint, while laterality, indication for placement (cosmetic vs reconstructive), and indications for CXR as secondary endpoints.

## Materials and Methods

This study classifies as a retrospective observation study, which was conducted in accordance with the Strengthening the Reporting of Observational Studies in Epidemiology (STROBE) checklist. It received ethics committee approval (Ref. 7001_2020) and was conducted between April 2020 and June 2021. The study consisted in recognizing the presence of breast implants on CXR to calculate breast implant prevalence. For this purpose, it was conducted in two phases: a preliminary followed by second phase.

### Preliminary Study Phase: Identification of Reviewers

This phase consisted in identifying individuals who could consistently and reliably recognize BIs on CXR using predetermined criteria described by de Boer et al. [12], which are given as follows: (1) projection lines that follow the contour of the BI within the breast; (2) calcifications along the periprosthetic capsule; and (3) the typical signs of a metal magnetized valve/port of the tissue expander. This phase was initiated by submitting a 3-hour tutorial, training session and test to eight potential reviewers to assess their sensitivity and specificity in recognizing BIs on CXR. *Sensitivity* was defined as the ability to accurately assess CXRs as positive for BI, while *specificity* was defined as the ability to correctly assess CXRs as negative for BI. The training session included the retrospective analysis of 180 CXR of patients who had previously undergone BI surgery (insertion, revision or removal) at our facility in the form of a standardized quiz. Out of 180 radiographs, 60 displayed one or more BIs, while 120 showed none. All CXRs were performed in our center using a remote-controlled direct radiology (DR) system and were collected through our facility’s Centricity Enterprise Web application PACS (Picture Archiving and Communication System) [GE Healthcare, USA]. Radiological images were assessed in dual-headed workstations, with dedicated NEC [Sharp NEC Display Solutions Ltd.] high-luminosity and high-resolution reporting monitors (2,5 K x 2 K), as per usual normal working conditions for radiologists. BI status (i.e., presence or absence) was confirmed by using our prospectively maintained breast reconstruction patients database with a minimum follow-up of 3 years. No medical file was directly accessed, to respect patient confidentiality. The eight potential reviewers all received the same instructions and training, and were later asked to test their skills by responding to a 180-question test using a sample answer sheet. Individual sensitivity and specificity were calculated upon completion, and the three most accurate participants were selected (Table 1).

**Table 1:** Sensitivity and specificity of the aspiring reviewers during the validation portion of the study, based on 180 chest X-rays

	A	B	C	D	E	F	G	H
Sensitivity (%)	96	91.7	90	90	87	81	68	57
Specificity (%)	98	98.3	97.5	92	98	96	98	90

### Secondary Study Phase: Assessment of bi Prevalence

This phase consisted in the large-scale evaluation of BI prevalence in the target population, which was the female population who received CXR at our institution. Inclusion criteria were as follows: women aged between 20 and 70, who received one or more CXRs in our facility, regardless of indication, between 01/01/2019 and 31/12/2019. Only one CXR per patient was included. Exclusion criteria were given as follows: pregnant woman and low-quality CXR. Any disagreement among reviewers was settled during consensus-based meetings, with blinded reevaluation.

An appointed third party was in charge of selecting patients in accordance with inclusion and exclusion criteria. They selected specific identifying codes of all eligible patients from the PACS and stored their radiological images, ensuring the full anonymity of each image before submitting them to the reviewers. All data were manually tabulated in an Excel spreadsheet (Microsoft Office, Albany, USA) later used for calculations. The estimated prevalence of BIs was calculated similarly to what was described in previous studies [12], with the following formula as a function of the presumed true prevalence (p), the specificity (spec) and the sensitivity (sens) of the reviewers:

$${\text{Estimated}}\;{\text{prevalence}} = \left( {1 {-}p} \right) \times \left( {1{-}{\text{spec}}} \right) \pm p \times {\text{sens}}$$

To determine secondary endpoints, the indication of each CXR was recorded by the third party before the anonymization process. Conversely, laterality and distinction between cosmetic or reconstructive BI indication were made by each reviewer, according to objective signs from the images, such as absence of breast gland and/or nipple, and marked as “unknown” in cases where it could not be determined. Mastectomies were distinguished from aplasia according to age, presence of central lines and eventual hemostatic clips. The homogeneity of age structures of the 4 populations (Sant'Andrea, Rome, Lazio, Italy) was assessed using the phi correlation coefficient, to verify whether data from the large population at a hospital-level could have significant difference with the population at a local, regional and national level.

## Results

### Preliminary Phase

Reviewers included two fully trained radiologists expert in breast imaging, two plastic surgery consultants, two plastic surgery residents, and two medical students without prior training (Table 1). Median sensitivity of the eight reviewers was 87.0% (range 57.0–96.0%), whereas median specificity was 97.0% (range 90.0–98.3%). The highest scores in terms of sensitivity and specificity were achieved by one radiologist (A), one plastic surgery consultant (B) and one plastic surgery resident (C), who later served for the following phase of the study.

### Secondary Phase

After initial assessment, 3537 unique patients aged 20–70 had undergone a CXR at our institution within the selected time frame: 82 (2.3%) were excluded due to artifacts or unreadable diagnostic images. Thus, 3448 CXR were deemed eligible and their images were analyzed. Each patient CXR had a posteroanterior and a latero-lateral view, except for the emergency radiographs, where a single view was taken in most instances. Disagreement was observed in 116 instances (3.4%), and all were solved after consensus-based discussion. Mean age was 52.5 years (ranging from 20 to 70), the population was subdivided into the following groups: 20- to 30-year-olds (246 patients), 31- to 40-year-olds (383 patients), 41- to 50-year-olds (759 patients), 51- to 60-year-olds (940 patients) and 61- to 70-year-olds (1120 patients). Since the sample sizes were very big, the p value is not indicated; therefore, measures of effect size (phi) were calculated. In this study, phi coefficient was next to zero: Sant'Andrea versus Roma: 0.021, Sant'Andrea versus Lazio: 0.018, Sant'Andrea versus Italia: 0.005, which led to the conclusion that the age distribution of the control populations does not significantly differ from the Sant'Andrea age distribution. In fact, with a sufficiently large sample, a statistical test will almost always demonstrate a significant difference, unless there is no effect whatsoever, that is, when the effect size is exactly zero, yet very small differences, even if significant, are often meaningless [14]. Differences in distribution of age populations across populations are detailed in Table 2. Indications for CXR are summarized in Table 3.

**Table 2:** Population distribution according to age range in the Sant’Andrea Hospital, Rome, the Lazio region and Italy.

Age group	Sant'Andrea hospital	Rome city	Lazio region	Italy
20-30 yo	246 (7,1%)	218.232 (15,1%)	297.816 (15,3%)	3.200.718 (16,3%)
31-40 yo	383 (11,1%)	251.861 (17,4%)	339.340 (17,5%)	3.383.620 (17,2%)
41-50 yo	759 (22.0%)	349.232 (24,2%)	462.474 (23,8%)	4.478.992 (22,8%)
51-60 yo	940 (27,2%)	360.455 (24,9%)	479.682 (24,7%)	4.757.609 (24,3%)
61-70 yo	1.120 (32,5%)	265.702 (18,4%)	364.414 (18,7%)	3.796.464 (19,4%)
Total	3.448 (100,0%)	1.445.482 (100,0%)	1.943.726 (100,0%)	19.617.403 (100,0%)

**Table 3:** Chest X-ray indications subdivided according to age ranges for the patients from the prevalence study

AGE RANGE	20–30 yo	31–40 yo	41–50 yo	51–60 yo	61–70 yo	Total
Pulmonary condition	75	83	138	177	200	673 (19.5%)
Cardiologic condition	50	107	166	153	108	584 (16.9%)
Preoperative assessment	34	85	242	315	472	1,148 (33.3%)
Postoperative assessment	5	12	29	49	79	174 (5.1%)
Post-midline insertion assessment	4	11	29	30	29	103 (3.0%)
Emergencies & End-stage malignancies	12	6	11	21	19	69 (2.0%)
Routine check-ups	33	44	67	89	87	320 (9.3%)
Unspecified	33	35	77	106	126	377 (10.9%)
Total	246 (7.1%)	383 (11.1%)	759 (22.0%)	940 (27.3%)	1120 (32.5%)	3,448 (100%)

**Fig. 1:**
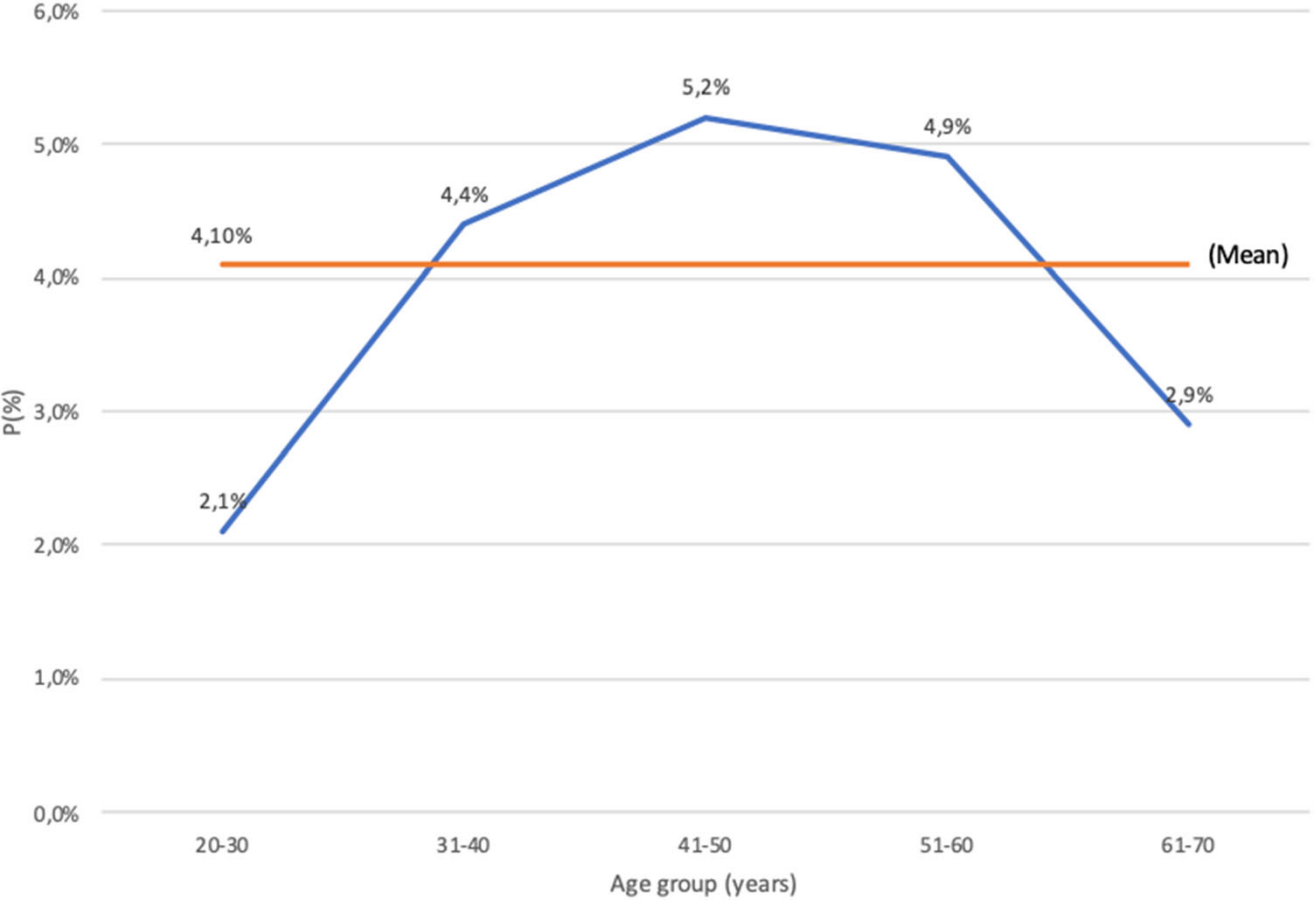
Estimated breast implant prevalence values among women between 20 and 70 years of age, subdivided into age groups and compared to the mean breast implant prevalence (P)

A total of 140 women were identified with BI, 106 (76%) bilateral while 34 (24%) unilateral, of which 14 (41%) to the right side and 20 (59%) to the left. Indication for BI placement was “unknown” in 18 (12.9%) women, while in the remaining 122 was cosmetic in 62 (50.8%) and reconstructive in 60 (49.2%) women. Overall mean BI prevalence was 4.1% and varied according to age range groups as 2.1% (20–30 age), 4.4% (31–40 age), 5.2% (41–50 age), 4.9% (51–60 age), and 2.9% (61–70 age) (Fig. 1).

## Discussion

**Fig. 2:**
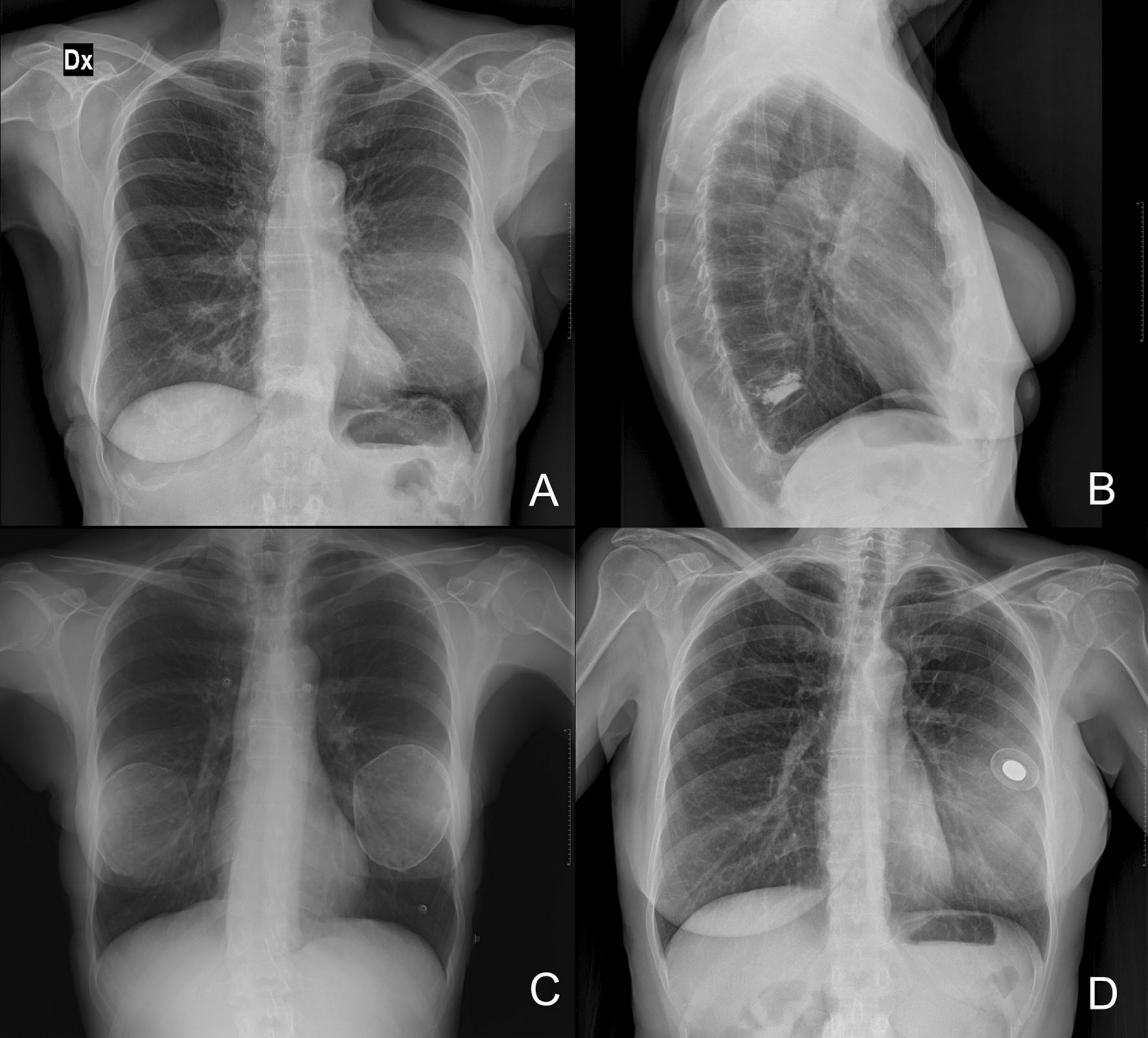
The de Boer et al. criteria for identifying breast implants on chest X-rays. They include projection lines that follow the contour of the implant within the breast, as seen on a posteroanterior (**A**) and latero-lateral view (**B**); calcifications along the periprosthetic capsule (**C**); and the metal magnetized valve/port of a tissue expander (**D**)

**Fig. 3:**
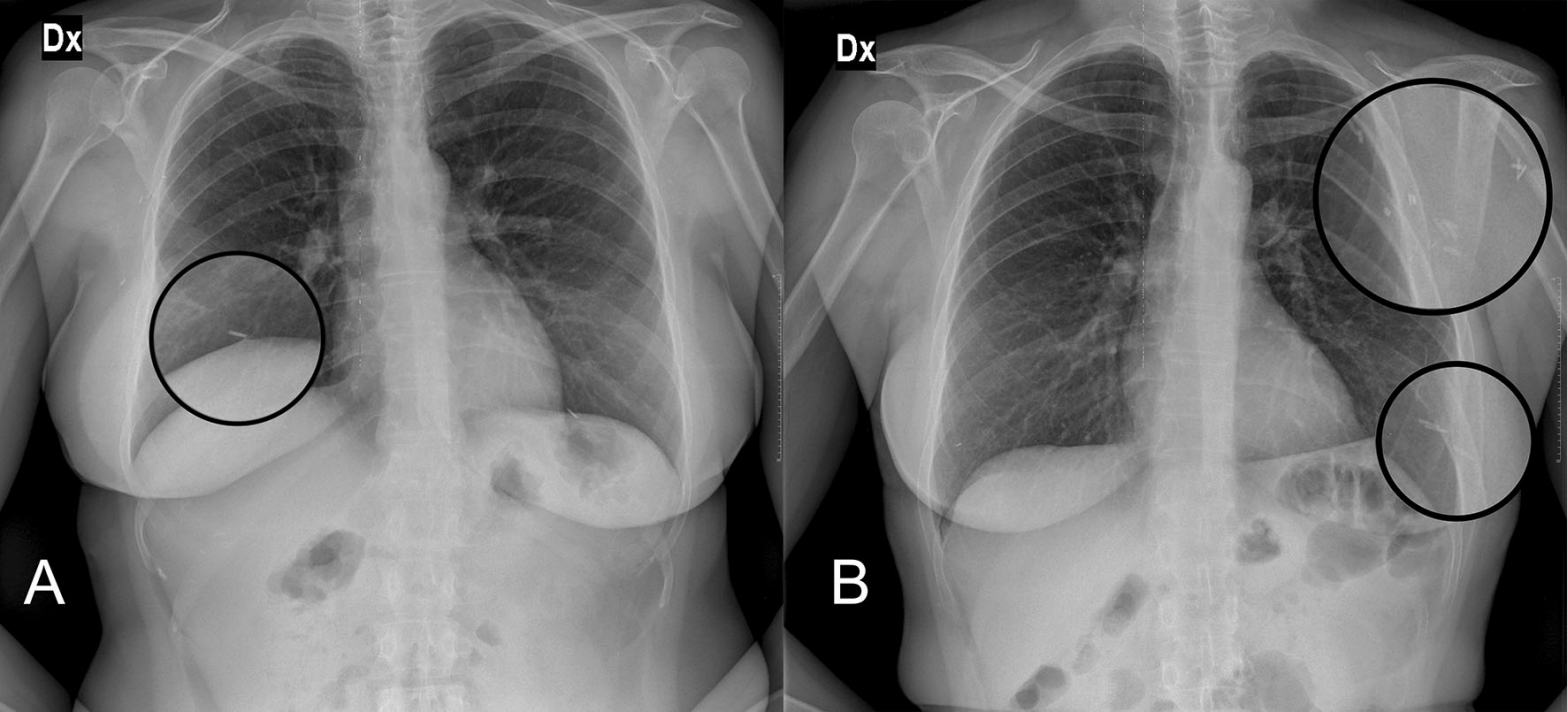
Differences between radio frequency identification (RFID) microchips bilaterally placed in breast implants **A** in a patient who underwent bilateral implant-based breast reconstruction, and hemostatic metal clips in the axilla and breast **B** in a patient who underwent left unilateral DIEP flap-based breast reconstruction

Determination of an accurate national BI prevalence is essential for the global health risk assessment. Being silicone radiopaque, the use of CXR on a wide population has emerged as a novel means for estimation of these data, since useful implant registries are missing [15]. Compared to de Boer’s used criteria [12], we added a fourth one which is the presence of radio frequency identification (RFID) transponder (Fig. 2). While implant projection lines were the most common sign to be found, calcifications could only be identified in older patients with a presumably longer time of implant permanence. Magnetized valves/ports were unsurprisingly recognized in only 7 patients (5%) because of the usually transient life of tissue expanders, and the fourth was exclusively associated with specific types of BIs using a novel non-invasive traceability system [16]. Although in breast reconstruction patients who received deep inferior epigastric perforator (DIEP) flap, the latter sign could be confused with a hemostatic metal clip, those are usually multiple, often located in the axilla as well as in linear fashion along the flap pedicle or sparse around the chest area. Conversely, the RFID transponder is single, oval-shaped, radiopaque structures, exclusively found in the region where the implant is located (Fig. 3).

**Fig. 4:**
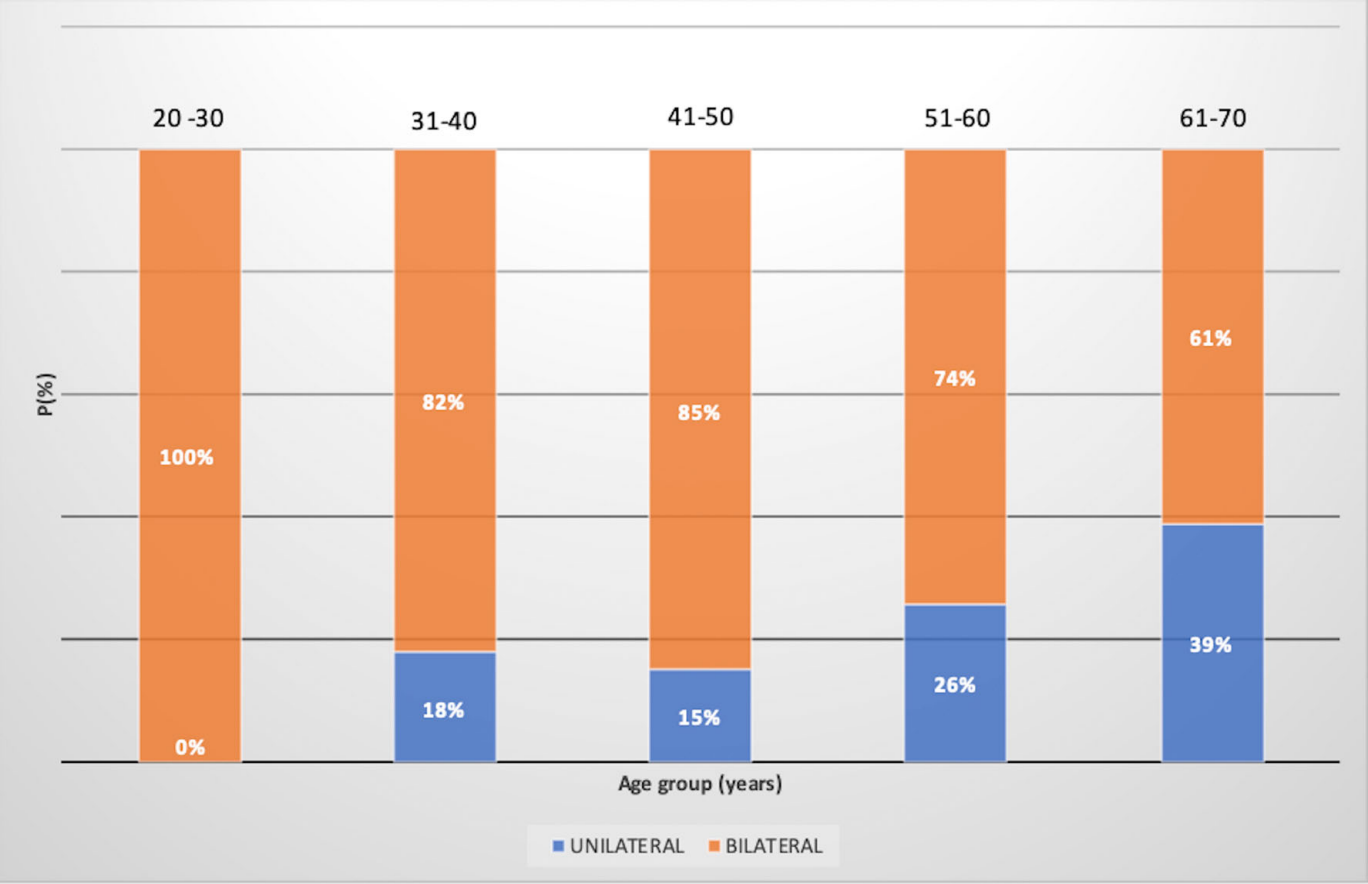
Breast implant laterality distribution per age group, in percentages. Patient population was subdivided into five age groups: 20–30, 31–40, 41–50, 51–60 and 61–70

Bilateral presence of BI varied across age groups, being higher in the younger population having more frequent cosmetic indication compared to the older having, mostly unilateral implants for reconstructive purposes (Fig. 4). The main limitation from our study is the fact that findings were only collected from a single, albeit large, medical center in Italy, potentially limiting the scalability of prevalence. However, our patient age distribution was compared to large epidemiological data on a regional and national scale, and was deemed statistically representative of Italy. Another limitation is how indication for implant placement could only be identified in 87.1% of patients due to the fact that it had to be determined according to indirect radiological signs.

The difference in BI prevalence compared to de Boer’s findings (respectively, 4.1% vs. 3.0%) may warrant some reflection on the heterogeneity of BI practices from country to country, suggesting a BI use more widespread in Italy than in the Netherlands. This can be confirmed by the International Society of Aesthetic Plastic Surgery (ISAPS) 2019 statistics, ranking Italy 5th worldwide for number of cosmetic breast augmentation surgeries (56,073 procedures) [13], just similar but behind USA, Brazil, Japan, and Mexico. While the Netherlands did not make the top sixteen countries worldwide and is only included in aggregate by ISAPS within Global estimates with 12,419 breast implant procedures, of which 8149 (66%) cosmetic augmentations and 4242 (34%) reconstructive [17]. The Italian female population counts 30.8 million while the Dutch 8.75 million, which means that 0.182% (56,073/30.8 million =) of Italian females vs 0.093% (8149/8.75 million =) of Dutch females received a breast augmentation in 2019 [18, 19]. While these figures only represent rough estimates, they suggest that Italian females underwent nearly twice as many breast augmentations per capita as Dutch females did. Unfortunately, a similar direct comparison between breast reconstruction cannot be made, as data are not readily available in Italy due to the lack of formal Breast Implant Registry, but a comparison can be extrapolated by breast Cancer data. In 2020, the Global Cancer Observatory reports 55,133 new breast cancer in Italy [20], counting 13.3% of all new cancers, while The Netherlands’ 15,725 counting 11.9% of all new cancers. Therefore, with a larger incidence of breast cancers in Italy, it is reasonable to infer that breast reconstruction figures are higher as well. These notions strengthen the importance of an Italian estimate to be adopted in countries with higher BI practice.

Finally, being our 4.1% figure representative of the Italian female population aged 20–70 (20,139,440) [21], we could presume 825,717 women with BI in Italy. As the rest of the European Union of 28 countries (EU-28), counting 170,611,364 women aged from 20 to 70, could not be considered all with high BI practice, neither with low, we could reasonably adopt a mean value of 3.55% prevalence rate, estimating 6.056.703 women with BI compared to 5,118,341 as by de Boer et al.’s calculation.

Their original study presents other differences to be considered compared to ours. In their preliminary phase, they recruited patients who had undergone a CT and/or MRI scan within ± 3 months from the CXR, selecting a brief time frame to minimize the risk that BI status might have changed between the scan and the radiograph. Differently we have selected CXR from patients with a known BI history, hence reducing the bias of uncertainty introduced by scans performed at a different time from the radiograph. Consequently, we found that our reviewers’ sensitivity (87.0%) and specificity (97.0%) exceeded de Boer et al.’s, who reported using a threshold of 70% for sensitivity and 80% for specificity to select theirs. Additionally, our study includes data collected from a large center in Central Italy, with a catch basin representing both rural and urban geographies, whereas de Boer et al. from 2 smaller regional centers in the Netherlands. Conversely, they used epidemiologic data from their Breast Cancer Screening Program to apply their local BI prevalence to the entire country. This was not feasible in Italy due to differences in data collection, and we compared our population with local, regional and national ones and found no significant differences in terms of homogeneity in age distribution.

It was notable that within this study, the mean age was sizably higher than in the Netherlands (52.5 vs. 46.5 years). This is likely not due to differences in life expectancy, which are comparable in both populations: 82.012 years for the Dutch [22], 83.198 for the Italian in 2019 [23], but more likely be due to dissimilar BI indication among both studies. We report that 47.1% of placements were cosmetic and 52.9% were reconstructive, while de Boer et al. found, respectively, 54.3% versus 45.7%. Breast augmentation patients have a lower mean age of 34 years [21], compared to 62 years in breast reconstructive patients [24–26]. Scarce presence of young individuals in the population studied is likely due to the fact that they require less frequently CXRs since they are generally fit and healthy [27]. This difference may indicate a possible underestimation of the cosmetic population as large-scale national multicenter data [28] and international findings [29] report that 75% of BI for cosmetic purposes while 25% reconstructive. Had our population included a breast augmentation-to-reconstruction ratio of 3:1, and had this ratio been confirmed for the Italian population, our BI prevalence could have been as high as 6.4%. We are unsure whether our percentage of younger participants was different from de Boer et al.’s because figures regarding age range distributions were not disclosed, thus could not be verified. These elements could explain the reported difference in BI placements, but also highlight the possibility of selection bias, which can only be solved by recruiting more patients from different hospitals in other parts of the country or even beyond national border. This is something we have already begun to work toward in the form of a multicentric study on a European scale.

Despite our best efforts, the use of unconventional methods for calculating BI prevalence will never surpass mandatory reporting in NBIRs [30]. Therefore, we strongly support recommendations of the Scientific Committee on Health, Environmental and Emerging Risks (SCHEER)’s to implement their use [2] for obtaining a better estimate of risks related to BI complications. In Italy, despite the implementation of Law n°86 on the 5th of June 2012 [31] for the creation of regional and national breast implant registries, nearly 10 years since the law’s enactment, the country’s tracking efforts are limited to a pilot study in 2019, with 269 participating surgeons as of September 2021: [32] a number far from the required standards to generate widely applicable data. Therefore, as long as relevant institutions cannot guarantee the establishment of reliable NBIRs, our method appears the most reliable, particularly if validated in countries with different demographic.

## Conclusion

Having an accurate breast implant prevalence within the general population informs regulatory efforts, complication risks, and patient informed consent. We applied an already established method to the epidemiologic setting of the Lazio region in Central Italy. The results derived from this study, first of its kind in Italy and largest to date, helped estimating a more accurate BI prevalence in females between 20 and 70 years of age, statistically representative of entire Italy. The utility of this study spreads well beyond its national borders as Italy represents the top 5th breast implant market worldwide. Our figure could serve as a new benchmark denominator for calculating BI-related health hazards and complications for countries with similar breast implant practice to Italy. This methodology will allow greater understanding of global BI epidemiology with nation-specific prevalences through a multicenter project already in its infancy stages.
